# CYP2C19 polymorphisms and outcomes of Escitalopram treatment in Brazilians with major depression

**DOI:** 10.1016/j.heliyon.2020.e04015

**Published:** 2020-05-30

**Authors:** Rodrigo Bernini de Brito, Paulo César Ghedini

**Affiliations:** a Department of Pharmacology, Laboratory of Biochemistry and Molecular Pharmacology, Institute of Biological Sciences, Federal University of Goiás, Goiânia, GO, Brazil; b Brain Institute, Bueno Medical Center, Goiânia, GO, Brazil

**Keywords:** Human genetics, Psychiatry, Behavioral medicine, Biological psychiatry, Depression, Psychopharmacology, Pharmacology, CYP2C19 polymorphisms, Escitalopram, Major depressive disorder

## Abstract

Escitalopram (ESC), a selective serotonin reuptake inhibitor indicated for the treatment of depression and anxiety disorders, is primarily metabolized by cytochrome P450 (CYP) 2C19, which is a highly polymorphic enzyme known to cause inter-individual differences in pharmacokinetics. We hypothesized that CYP2C19 polymorphisms are associated with major depressive disorder (MDD) remission in patients treated with ESC in the long term. Thirty-one patients with MDD receiving chronic treatment with ESC monotherapy or combination therapy with other antidepressants (mirtazapine and bupropion), in naturalistic conditions, were included in the study. For comparison of genotype and phenotype frequencies, a group of 126 healthy subjects was also included. The CYP2C19∗2, CYP2C19∗3, and CYP2C19∗17 polymorphisms were analyzed by RFLP-PCR genotyping. The CYP2C19 genotypes and phenotypes were similar in patient and healthy subject groups. Four phenotypes were found in the healthy subject group: ultra-rapid (UM; 28%), extensive (EM; 52%), intermediate (IM; 17%), and poor metabolizers (PM; 3%). The patient group showed the UM (22.5%), EM (55%), and IM (22.5%) phenotypes. The UM patients had significantly higher ESC doses than both EM and IM patients (20.7 ± 4.5, 15.7 ± 3.8, and 14.0 ± 3.3 mg/day, respectively; p = 0.0041). Furthermore, all patients using ESC in combination with mirtazapine or bupropion antidepressants (ESC plus mirtazapine or bupropion) were UM metabolizers, suggesting that the ∗17 ultra-rapid allele seems to be the factor responsible for lower response to ESC, even at higher doses. The CYP2C19 UM phenotype is associated with higher ESC doses and antidepressant combinations for symptom remission in MDD patients.

## 1 Introduction

Major depressive disorder (MDD) is a mental disorder associated with an elevated risk of suicide and with the onset, persistence, and severity of a wide range of chronic physical disorders [1]. Antidepressant medications remain a key mode of treatment for moderate to severe MDD. However, their efficacy is variable and incomplete because 60–70% of depressed patients do not experience remission, and 30–40% of depressed patients do not show a significant response or experience drug side effects that result in treatment discontinuation, non-adherence, and chronic illness [2, 3]. Individual variability in the activity of drug-metabolizing enzymes is a major source of the differences to drug exposure and, hence, a contributor to variations in drug responses [4]. Therefore, potential biomarkers, such as genetic polymorphisms of cytochrome P450 (CYP) families, have been investigated as treatment response predictors for several antidepressants [5].

Escitalopram (ESC) is one of the most commonly prescribed selective serotonin reuptake inhibitors (SSRIs) used for the treatment of both depression and anxiety disorders [6], and is mainly metabolized by CYP2C19 [7]. Genetic variations in the *CYP2C19* gene may lead to changes in the metabolic activity of the CYP2C19 enzyme (increased or reduced function). CYP2C19∗1 is the wild-type allele that encodes a fully functional enzyme, whereas most of the CYP2C19 poor metabolizers are carriers of the variant alleles ∗2 and ∗3; the ∗17 variant is associated with increased enzyme activity [8]. These polymorphic variants are associated with different phenotypes, including extensive metabolizers (EM; CYP2C19∗1/∗1), intermediate metabolizers (IM; CYP2C19∗1/∗2, ∗1/∗3, or ∗2/∗17), poor metabolizers (PM; CYP2C19∗2/∗2, ∗2/∗3, or ∗3/∗3), and ultra-rapid metabolizers (UM; CYP2C19∗17/∗17 or ∗1∗17) [9, 10, 11, 12].

Although researchers found a significant relationship between CYP2C19 genotypes and serum concentrations of ESC, an association between genotypes and antidepressant treatment response was not observed in patients taking ESC for 12 weeks [5]. A study conducted for 8 weeks and assessing treatment response and adverse events due to ESC in patients with MDD did not find an association with the CYP2C19 phenotypes [2]. In another study, a correlation between CYP2C19 PM and early treatment response for ESC was verified in patients with panic disorder [11]. More recently, a study performed in a large patient population and using EM as a control group for comparison showed that ESC serum concentrations were significantly increased, 3.3-fold in PM and 1.4-fold in IM, and decreased by 10%–20% in UM. In addition, switching from ESC to another antidepressant within 1 year was approximately 3.0 times more frequent in the PM and UM groups [13]. Knowing that CYP2C19 polymorphisms can affect ESC efficacy and safety, the Clinical Pharmacogenetics Implementation Consortium (CPIC) Guideline provides ESC dosing recommendations for CYP2C19 genotypes [12].

Based on the information mentioned above and considering that the impact of CYP2C19 polymorphisms on the long-term treatment response to ESC is not clear, we explored the association between CYP2C19 metabolizers and remission of depressive symptoms in a group of MDD patients receiving ESC for the long term (more than 3 years).

## 2 Material and methods

### 2.1 Patient population

Our naturalistic and observational clinical study enrolled 31 patients from the Brain Institute in Goiânia, Goiás State, Brazil. We collected the social and demographic information of consecutively recruited male and female patients ranging in age from 18 to 65 years (age mean 49.0 ± 13.9 years). The criteria for including patients with MDD was defined by the Diagnostic and Statistical Manual of Mental Disorders (5^th^ ed.; DSM-5). All patients started the treatment with ESC monotherapy, however, those patients who did not show symptoms remission received an adjuvant antidepressant. These subjects met the criteria for the remission of MDD for at least 12 months; they had received ESC for the long term and had no history of dropout from or non-compliance with the depression treatment. Subjects were receiving ESC (10–30 mg per day) in monotherapy or combination with mirtazapine or bupropion (ESC combination). The remission of depression symptoms was assessed by measures of the Hamilton Depression Rating Scale (HDRS) [14]; remission was considered for a score ≤7. The collection of biological material was performed only in patients who were in symptom remission for at least 12 months with the treatment. The data for each patient's HDRS were collected and analyzed as recorded in his or her file. The values at the baseline of the treatment with ESC were compared with those when genotype testing was performed.

Patients were ineligible if they were pregnant or lactating women; if they were younger than 18 years old; if they had received primary or comorbid diagnoses of schizophrenia, schizoaffective disorder, bipolar disorder, dementia, or clinically significant medical disorders; if they had received abnormal laboratory results at screening; or if they had an alcohol or substance dependence, based on DSM-5 criteria.

A total of 126 healthy individuals of both sexes (71 men and 55 women), aged between 19 and 73 years (mean 30.0 ± 8.6 years), selected from the general population and without a history of drug abuse and psychiatric or psychotic disorders including schizophrenia, bipolar disorder, and major depression, were enrolled as a control group. Data on healthy volunteers were obtained using a self-report questionnaire, including demographic characteristics, eating habits, mental and clinical diseases and drugs used.

All experimental procedures and protocols were approved by the local Ethics in Research Committee (Protocol CEP/UFG 204/2009). Written informed consent was obtained from all participants.

### 2.2 DNA genotyping

DNA was extracted from the venous blood of each patient with a Purelink Genomic DNA Mini Kit® (Invitrogen, San Diego, USA); the manufacturer's instructions were strictly followed. Genotyping was performed using a polymerase chain reaction (PCR) followed by single primer extension. The details of amplification and PCR product characteristics of CYP2C19∗2 (rs4244285; c.681G > A) CYP2C19∗3 (rs4986893; c.636G > A), and CYP2C19∗17 (rs12248560; c.-806C > T) are presented in Table 1. The primers and nested PCR protocol for CYP2C19∗17 were performed according to Anichavezhi et al. [15]. The DNA fragments resulting from PCR and PCR-RFLP were analyzed on ethidium bromide-stained agarose.

**Table 1:** Details of product PCR size for CYP2C19∗2, CYP2C19∗3, and CYP2C19∗17 PCR and RFLP characteristics. CYP2C19∗17 analyses consisted of a nested PCR (PCR 1 and PCR 2).

Allele	PCR product size (bp)	Enzyme	Wild-type (size bp)	Heterozygous (size bp)	Homozygous mutant (size bp)	Forward primer (5’ – 3′)	Reverse primer (5’ – 3′)
2C19∗2	223	SmaI	113–110	223-113-110	223	CAACCAGAGCTTGGCATATTG	TAAAGTCCCGAGGGTTGTTG
2C19∗3	271	BamHI	175–96	271-175-96	271	AAATTGTTTCCAATCATTTAGCT	ACTTCAGGGCTTGGTCAATA
2C19∗17 (PCR 1)	470	---	---	---	---	GCCCTTAGCACCAAATTCTC	ATTTAACCCCCTAAAAAAACACG
Nested (PCR 2)	143	NsiI	166–27	143-116-27	143	AAATTTGTGTCTTCTGTTCTCAATG	AGACCCTGGGAGAACAGGAC

The classification of CYP2C19 metabolizer phenotypes used in this study followed the standardized terms for clinical pharmacogenetic test results recommended by CPIC [16].

### 2.3 Statistical analysis

Tests for Hardy–Weinberg equilibrium and linkage disequilibrium were performed using Arlequin version 3.5.1.2. Group comparisons were performed using χ^2^ and Fisher's exact test for categorical variables, Kruskal–Wallis and Mann–Whitney U tests for nonparametric continuous variables, and Student's T test or analysis of variance (ANOVA) for continuous data. A p-value of less than 0.05 was considered statistically significant. Statistical analyses were performed using the SPSS software package (version 21.0; SPSS Inc., IL, USA).

## 3 Results

### 3.1 Distribution of the CYP2C19 genotypes in healthy subject and patient groups

The CYP2C19 allele and genotype frequencies were similar in healthy subject and patient groups (p = 0.1238). The proportions of allele frequencies are shown in Table 2. The proportion of the wild-type CYP2C19∗1 allele (69.1%) was significantly higher than that of the other alleles (∗2, ∗3, and ∗17). The predominant variant allele was CYP2C19∗17 (17.2%) followed by CYP2C19∗2 (12.7%) and CYP2C19∗3 (1.0%). The genotype frequencies in healthy subjects and patients are shown in Table 3. The CYP2C19 genotype frequencies were consistent with the Hardy–Weinberg equilibrium (χ^2^ = 3.28, p = 0.07). The proportions of EM, IM, and UM were not different between groups (p = 0.5189, 0.1367 and 0.5581, respectively), and the PM phenotype was not found in the patient group.

**Table 2:** CYP2C19 allele frequencies in healthy subjects (71 men and 55 women) and patients (9 men and 22 women).

	Alleles				
	∗1	∗2	∗3	∗17	p-value
Healthy subjects [n (%)]	175 (69.4)	30 (11.9)	3 (1.2)	44 (17.5)	0.1238
Patients [n (%)]	42 (67.8)	10 (16.1)	---	10 (16.1)	
Total [n (%)]	217 (69.1)	40 (12.7)	3 (1.0)	54 (17.2)	

p-values were obtained using nonparametric Kruskal–Wallis test.

**Table 3:** Comparison of different phenotypes in healthy subjects and patients.

Phenotype	Genotype	Healthy subjects (n = 126)	Patients (n = 31)	p-value
Extensive metabolizers	CYP2C19∗1/∗1	62 (49%)	14 (45%)	0.5189
Intermediate metabolizers	CYP2C19∗1/∗2	19 (15%)	7 (22.6%)	0.1367
	CYP2C19∗1/∗3	2 (1.6%)	-	
	CYP2C19∗2/∗17	4 (3.2%)	3 (9.7%)	
Poor metabolizers	CYP2C19∗2/∗2	3 (2.4%)	-	nd
	CYP2C19∗2/∗3	1 (0.8%)	-	
Ultra-rapid metabolizers	CYP2C19∗1/∗17	30 (24%)	7 (22.6%)	0.5581
	CYP2C19∗17/∗17	5 (4%)	-	

Table showing categorization of study subjects into different groups based on their genotype [9, 10, 11, 12].

p-values were obtained using chi-square test; p > 0.05 was considered to be significant with a two-tailed test.

### 3.2 Demographic-clinical characteristics

The study included 31 patients with MDD (22 women and 9 men), receiving treatment with ESC alone or in combination with other antidepressants. Correlation of treatment response with CYP2C19 phenotypes is shown in Table 3. The patients had received ESC for a mean of 3.48 ± 1.4 years; they had no or little alcohol use, were not smokers, and did not use concomitant herbal medicines. At the baseline ESC treatment, the HDRS scores for each phenotype group were 23.7 ± 3.5 (EM), 22.4 ± 3.5 (IM) and 25.1 ± 4.4 (UM). After treatment (ESC alone or ESC combination), at genotype testing, all groups presented symptoms remission with HDRS scores of 3.6 ± 1.5 (EM); 2.7 ± 1.4 (IM) and 2.6 ± 1.2 (UM) (Table 4).

**Table 4:** Clinical characteristics for the phenotype in the patient group.

	EM	IM	UM	p-value
Male/female (N)	4/10	2/8	3/4	0.650
Dosage mg/day (SD)	15.7 ± 3.8	14.0 ± 3.2	20.7 ± 4.5∗	0.0041
HDRS baseline, mean ± SD	23.7 ± 3.5	22.4 ± 3.5	25.1 ± 4.4	0.001
HDRS at genotype testing, mean ± SD	3.6 ± 1.5	2.7 ± 1.4	2.6 ± 1.2	
Time receiving ESC (years), mean ± SD	3.1 ± 1.5	3.3 ± 1.5	3.4 ± 1.3	0.8987
ESC combination (N)	0	0	7	0.0001^ϯ^

∗ANOVA with Bonferroni adjustment.

^Ϯ^chi-square test for trend.

## 4 Discussion

Although the association between CYP2C19 polymorphisms and ESC efficacy and safety has been well studied among multiple populations, to our knowledge, this is the first study showing the association between CYP2C19 polymorphisms and ESC long-term treatment responses in Brazilian patients with MDD.

The genotype and allele frequencies were similar between the healthy subject group and the patient group and are in agreement with what is expected for the Brazilian population, the ∗17 allele being more prevalent than ∗2 and ∗3 variants, respectively [10, 17, 18].

According to genotype, we found the phenotype for three types of metabolizer for CYP2C19 in the patient group: UM, EM, and IM. Based on this information, it was possible to observe that the MDD remission in the UM group (∗1/∗17) was achieved with ESC in combination with other drugs (mirtazapine or bupropion). The UM phenotype has been associated with lower serum concentrations of ESC, which might imply an increased risk of therapeutic failure [19]; it has been suggested to monitor plasma concentration and titrate the dose to a maximum of 150% in response to efficacy and adverse drug events or to select an alternative drug in CYP2C19 UM patients taking ESC [20]. According the CPIC Guideline, because there are insufficient data to calculate an initial ESC dose for UM, an alternative SSRI not extensively metabolized by CYP2C19 may be used, if deemed appropriate given other medications and clinical considerations [12].

In this study, despite the greater ESC dosage in the UM group, this group only achieved remission of depression symptoms using a pharmacological combination of ESC with another antidepressant. For the EM and IM groups, monotherapy with ESC was sufficient for the remission of symptoms. It is possible to suggest that the increase of ESC dosage in the UM patients was not sufficient (mean 38% higher than EM and IM patients) and, for this reason, the combination with another antidepressant was necessary to achieve remission of depression symptoms. Although the CPIC Guideline for CYP2C19 genotypes and dosing of ESC for UM patients recommends considering an alternative drug to ESC, which is not predominantly metabolized by CYP2C19 [12], the patients studied here showed therapeutic efficacy using ESC associated with another antidepressant. However, considering there were no EM and IM patients with combined therapy to compare, our finding should be replicated in larger-scale studies before any clinical implications are considered. In addition, the CPIC Guideline recommendations should always be applied.

This study has some limitations. First, the sample size of patients analyzed was small. Second, we did not measure the plasma concentration of ESC, although the higher doses that we found in the UM individuals is in accordance with other studies [19, 20]. Third, an important limitation of the study is the single-gene analysis, since ESC is also metabolized by CYP2D6 and CYP3A4 enzymes and transported by ABCB1, which may also be used to predict patient ESC treatment outcomes based on multiple gene polymorphism analysis [21, 22].

## 5 Conclusion

This study demonstrates that Brazilian CYP2C19 UM patients require higher ESC doses to achieve MDD symptom remission. This finding should be validated in larger-scale studies and *CYP2D6*, *CYP3A4*, and *ABCB1* added for a complete analysis.

## Declarations

### Author contribution statement

R. de Brito: Conceived and designed the experiments; Performed the experiments; Analyzed and interpreted the data; Wrote the paper.

P. Ghedini: Conceived and designed the experiments; Performed the experiments; Analyzed and interpreted the data; Contributed reagents, materials, analysis tools or data; Wrote the paper.

### Funding statement

This work was supported by 10.13039/501100005285Fundação de Amparo à Pesquisa do Estado de Goiás (Fapeg; grant number 200910267000499) and 10.13039/501100003593Conselho Nacional de Desenvolvimento Científico e Tecnológico (CNPq; grant number 311364/2017-9).

### Competing interest statement

The authors declare no conflict of interest.

### Additional information

No additional information is available for this paper.
